# 

**Published:** 1895-06

**Authors:** 


Secretary.

---

### REUNION OF GRADUATES OF THE AMERICAN VETERINARY COLLEGE.

DEAR DOCTOR: A reunion of the American Veterinary College graduates now in Pennsylvania will be held at Lansdowne, Delaware County, Tuesday, June 11, 1895, at 4 o'clock. Trains leave Broad and Market Streets at 3.18 and 3.47. Lunch will be served. The encouragement we have received throughout the State warrants us in expecting a well-attended meeting and trust that you may be one of the number, as the State Alumini Association, started in February, will be placed in more definite shape, and officers for the ensuing year will be elected.

The future of veterinary medicine depends largely upon the closer affiliation of the veterinary practitioners, and this is accomplished best through closer associations of the alumni, and we hope the American Veterinary College may in the future, as in the past, maintain the lead among veterinary colleges.

A meeting of the Keystone Medical Veterinary Association will be held in the evening, and we sincerely hope that all will remain and attend it.

Hoping to have the pleasure of meeting you at that time,

I am fraternally, W. L. RHOADS,

LANSDOWNE, PA. Secretary.

### U. S. V. M. A.

In connection with the coming meeting of the U. S. V. M. A. a party of twenty-five will be made of veterinarians from New York, New Jersey, Pennsylvania, and Maryland to go to Des Moines in September next. A special Pullman sleeping-car for the party will be secured, and reduced rates have been obtained. It will be taken out by the Baltimore and Ohio Railroad. At Chicago the only change of cars will be made. Veterinarians wishing to avail themselves of a place with this party can obtain all information as to rates, etc., by addressing W. Horace Hoskins, 3452 Ludlow Ptreet, Philadelphia.

Chairman Trumbower, of the Committee on Diseases, promises to give the best and most complete report ever enjoyed by the Association. All his colleagues on the Committee have been assigned special topics, and the Chairman will present important matters for consideration.

Dr. Tait Butler will read a paper entitled "Accidents Incident to the Casting and Confining of Animals for Surgical Operations." This subject will surely prove interesting and instructive to every veterinarian present.

The California State Veterinary Medical Association at its June meeting will elect a delegate to the coming meeting in Des Moines. California veterinarians are enthusiastic over the prospects of a meeting on the Pacific Coast in 1897.

### PERSONAL.

Dr. Clyde E. Fouse, a recent graduate of the Veterinary Department of the University of Pennsylvania, is now associated with the teaching staff of that Department as Assistant Demonstrator of Anatomy.

Dr. Thomas Young, a well-known veterinary surgeon of Delaware County, Pa., has just received the nomination for Prothonotary of that county. This is one of the leading county offices.

Dr. Arthur Salinger, a graduate of the Veterinary Department of the University of Pennsylvania, the coming college year, will fill the position of Demonstrator of Veterinary Surgery.

Dr. W. B. E. Miller's many friends will be glad to learn of his promised convalescence from the painful injuries recently received while in the act of extracting a molar tooth from a mule.

Prof. Dewar has been appointed Principal of the Royal (Dick) Veterinary College, after an acrimonious discussion and severe criticism of the Town Council, under whose jurisdiction lies the application of the Dick Trust.

Among the veterinarians whose names are already being mentioned for places on the State Veterinary Medical Examining Board of Pennsylvania are those of Drs. Harger, Hoskins, John R. Hart, and W. T. S. Werntz, of Philadelphia; Dr. Conard, of West Grove; Dr. Lusson, of Ardmore; Dr. Weber, of Lancaster; Dr. Sallade, of Pottsville; Dr. Walters, of Wilkesbarre; and Dr. J. C. McNeill, of Pittsburg. Five are to be appointed.

Dr. W. C. Fair, of Cleveland, Ohio, recently addressed the Master Horseshoers' Association at their convention in that city.

Dr. E. B. Ackerman officiated as veterianarian to the recent Brooklyn horse-show.

Dr Richard Middleton, a graduate of the American Veterinary College, after pursuing a course of dentistry at the University of Pennsylvania, has embarked for Germany, where he proposes to practise his newly adopted vocation.

Drs. Zuill, Pearson, and Harger officiated as veterinarians to the Philadelphia horse-show recently held at Wissahickon Heights.

Dr. John Airth, connected with the meat-inspection department of the Bureau of Animal Industry, has been transferred from Sioux City, Iowa, to Kansas City, Missouri.

Dr. Daniel Le May, who has recently been stationed at the United States Cavalry and Light Artillery School, Ft. Riley, Kansas, has been transferred to Ft. Grant, Arizona.

Dr. M. A. Piche, veterinarian of 1st Cavalry of United States, has been transferred, by a change of location of his regiment, from Ft. Grant, Arizona, to Ft. Riley, Kansas.

Dr. Tait Butler, in his Southern field of work, has found it necessary to give much of his time to the pioneer work of first encouraging the growth of the live-stock industry. He has already taken the preliminary steps toward organizing a State Stock-breeders' Association, the establishing of an agricultural paper, and the breeding of live-stock.

